# Supplementary material for: Enhanced propagation of motile bacteria on surfaces due to forward scattering
Source: Nat Commun. 2019 Sep 11;10:4110. doi: 10.1038/s41467-019-12010-1 (PMC6739365; doi:10.1038/s41467-019-12010-1)
Supplement: Supplementary file 1 — Supplementary Information [file 41467_2019_12010_MOESM1_ESM.pdf]

Supplementary Information for “Enhanced propagation of motile bacteria on surfaces due to forward scattering”

Makarchuk *et al.*

SUPPLEMENTARY FIGURES

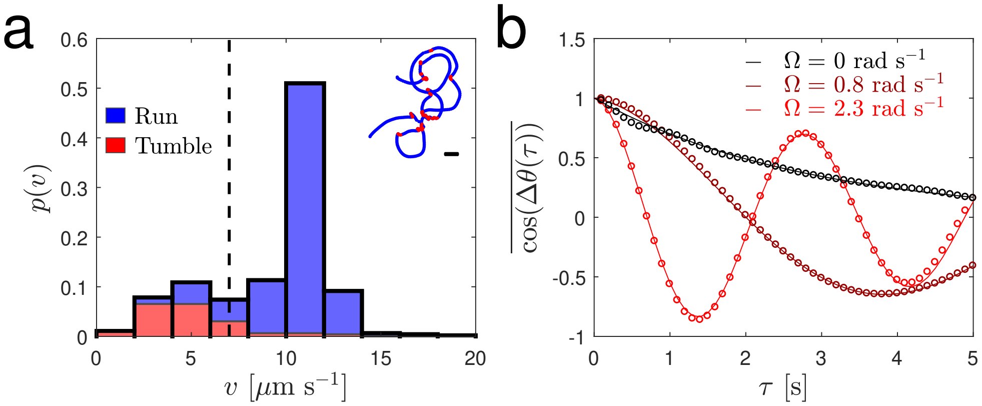

Supplementary Figure 1. Estimation of the *E. coli* translational and angular speeds in the absence of obstacles. (**a**) Probability distribution $p(v)$ of the cell's instantaneous speed v for the trajectory shown as inset. Both trajectory and distribution are divided in the two contributions from the run phase (blue) and the tumble phase (red) (Methods). The part of the distribution to the right of the threshold (vertical dashed line) provides the cell's average translational speed (here $10.3\,\mu\mathrm{m\,s^{-1}}$). The black scale bar in the inset corresponds to $10\,\mu$m. (**b**) Time decorrelation $\overline{\cos(\Delta\theta(\tau))}$ (circles) of the direction of motion of individual bacterial trajectories for different angular speeds Ω ($\Omega = 0\,\mathrm{rad\,s^{-1}}$, $\Omega = 0.8\,\mathrm{rad\,s^{-1}}$ and $\Omega = 2.3\,\mathrm{rad\,s^{-1}}$) estimated by fitting the experimental data to the function $f(\tau) = \cos(\Omega\tau)e^{-\tau/\tau_0}$ (solid lines) (Methods).

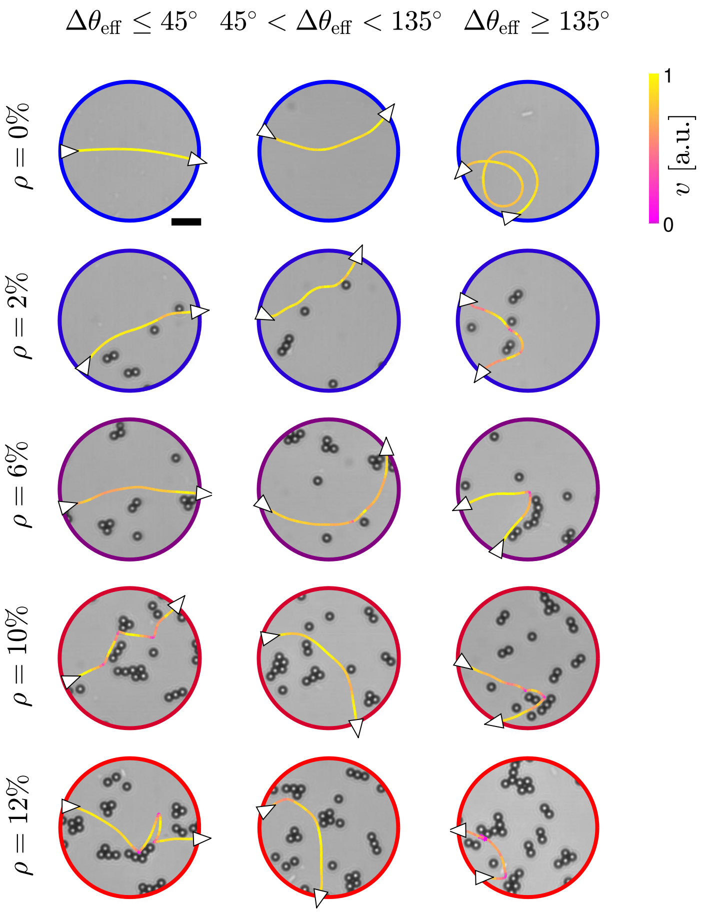

Supplementary Figure 2. Trajectories of *E. coli* cells swimming near surfaces with micro-obstacles. Exemplary trajectories of *E. coli* cells propagating through a circular area of radius $R = 25\,\mu$m for different obstacle densities ρ. For every value of ρ, three trajectories are shown for $\theta_{\mathrm{eff}} \leq 45°$, $45° < \theta_{\mathrm{eff}} < 135°$ and $\theta_{\mathrm{eff}} \geq 135°$ respectively. The white triangles on the trajectories represent the direction of motion when entering and exiting the circular area, while the trajectories' colour code represents the cells' instantaneous velocity v normalised to its maximum value. The black scale bar corresponds to $10\,\mu$m.

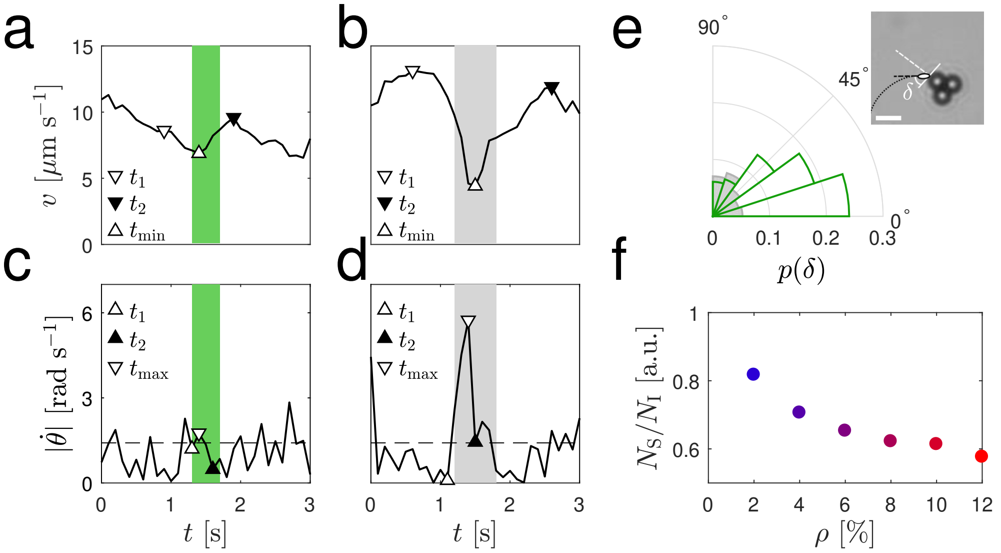

Supplementary Figure 3. Differences between cell-obstacle interactions classified as forward scattering and tumble-collisions. (**a-b**) Instantaneous velocity v and (**c-d**) absolute value of the instantaneous change in the direction of motion $|\dot{\theta}|$ for the trajectories shown in (**a,c**) Fig. 3a as example of forward scattering and (**b,d**) Fig. 3b as example of tumble-collision. The duration of the cell-obstacle interaction is highlighted by the shaded areas. The dashed horizontal lines in (**c,d**) highlight the extent of the change in direction of motion due to Brownian fluctuations for *E. coli* cells [1]. For the two quantities, the positions of the minima (upward-pointing triangles) and maxima (downward-pointing triangles) used to classify the cell-obstacle interactions as tumble-collisions or forward-scattering events are also shown (Methods). (**e**) Probability density distributions of the angle of approach δ for forward scattering (empty histogram) and tumble-collisions (filled histogram). As shown in the inset, δ is defined as the angle between the cell's direction of motion and the tangent to the obstacle at the start of the cell-obstacle interaction. The distributions are normalised to the total number of interactions to show the relative weight between forward-scattering events and tumble-collisions. The white scale bar in the inset corresponds to 5 μm. (**f**) Number of forward-scattering events N_S normalised to the total number of cell-obstacle interactions N_I as a function of obstacle density ρ.

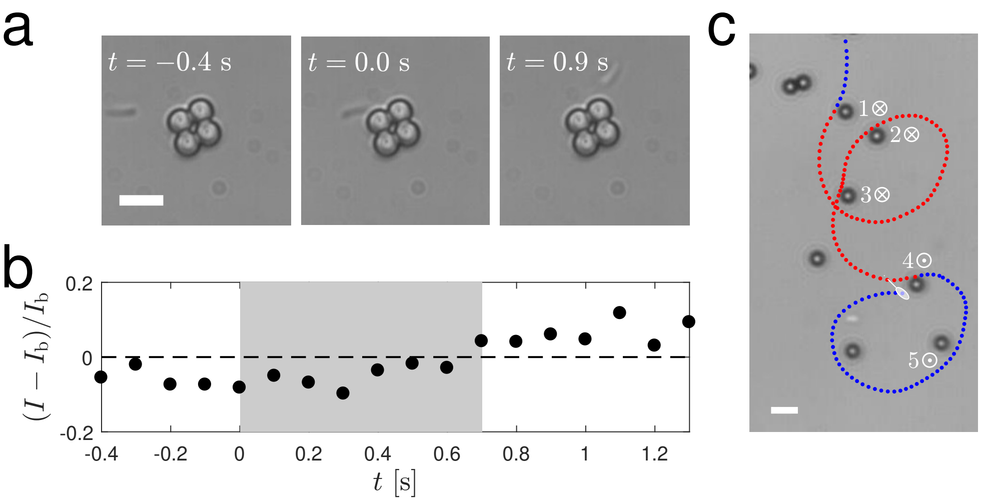

Supplementary Figure 4. **Change of *E. coli* cells' distance from the surface after forward scattering.** (**a**) Time lapse sequence of an *E. coli* cell swimming near an obstacle on the sample chamber's bottom surface during a forward-scattering event. This sequence was acquired using a 40x microscope objective (NA = 0.75, Leica HCX PL Fluotar). The cell-obstacle interaction starts at $t = 0\,\mathrm{s}$. The white scale bar corresponds to $5\,\mu\mathrm{m}$. (**b**) Relative change in the average gray-scale intensity I of the cell image with respect to the background value I_b (dashed horizontal line). As soon as the cell has crossed the obstacle, its distance from the surface changes as qualitatively highlighted by the fact that I goes from being darker than the background to being brighter. The gray shaded area highlights the duration of the cell-obstacle interaction. (**c**) Exemplary trajectory showing 5 forward-scattering events, where the stylised cell represents the trajectory's final position and direction of motion: initially, the cell is near the sample chamber's top surface as shown by the fact that it appears to swim clockwise (blue) in our setup; the cell's distance from the surface changes every time it passes an obstacle from its side where the consequent hydrodynamic torque points the cell towards the opposite surface, i.e upwards when near the bottom surface (4) and downwards when near the top surface (1); the cell's distance from the closer surface does not change otherwise (2,3,5). When the swimming cell changes surface of the sample chamber, the sign of the trajectory's chirality switches from clockwise (blue) to counterclockwise (red), and vice versa. The white scale bar corresponds to $5\,\mu\mathrm{m}$.

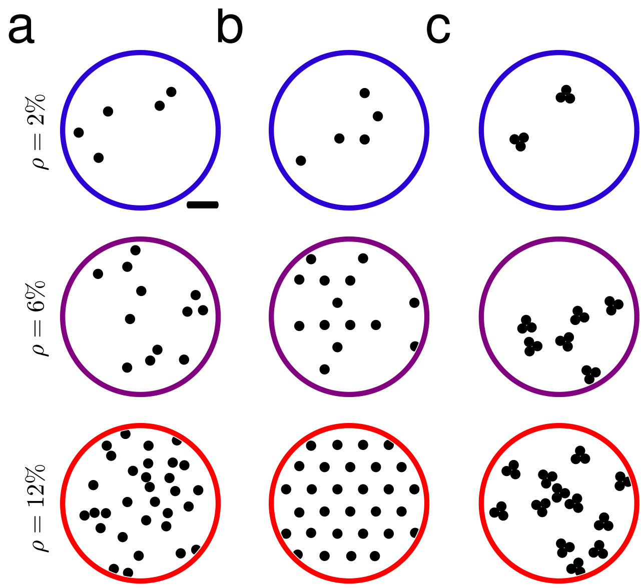

Supplementary Figure 5. **Simulated distributions of obstacles.** (**a-c**) Examples of different simulated obstacle distributions in a circular area of radius $R = 25\,\mu$m for increasing obstacle densities ρ (Methods). Individual obstacles are deposited (**a**) sequentially at random without overlap, (**b**) according to a periodic lattice and (**c**) sequentially as non-overlapping trimers (i.e. triangular clusters of obstacles) with a random orientation. In **b**, $\rho = 12\%$ corresponds to a complete lattice and lower obstacle densities are obtained by removing particles at random. The black scale bar corresponds to $10\,\mu$m.

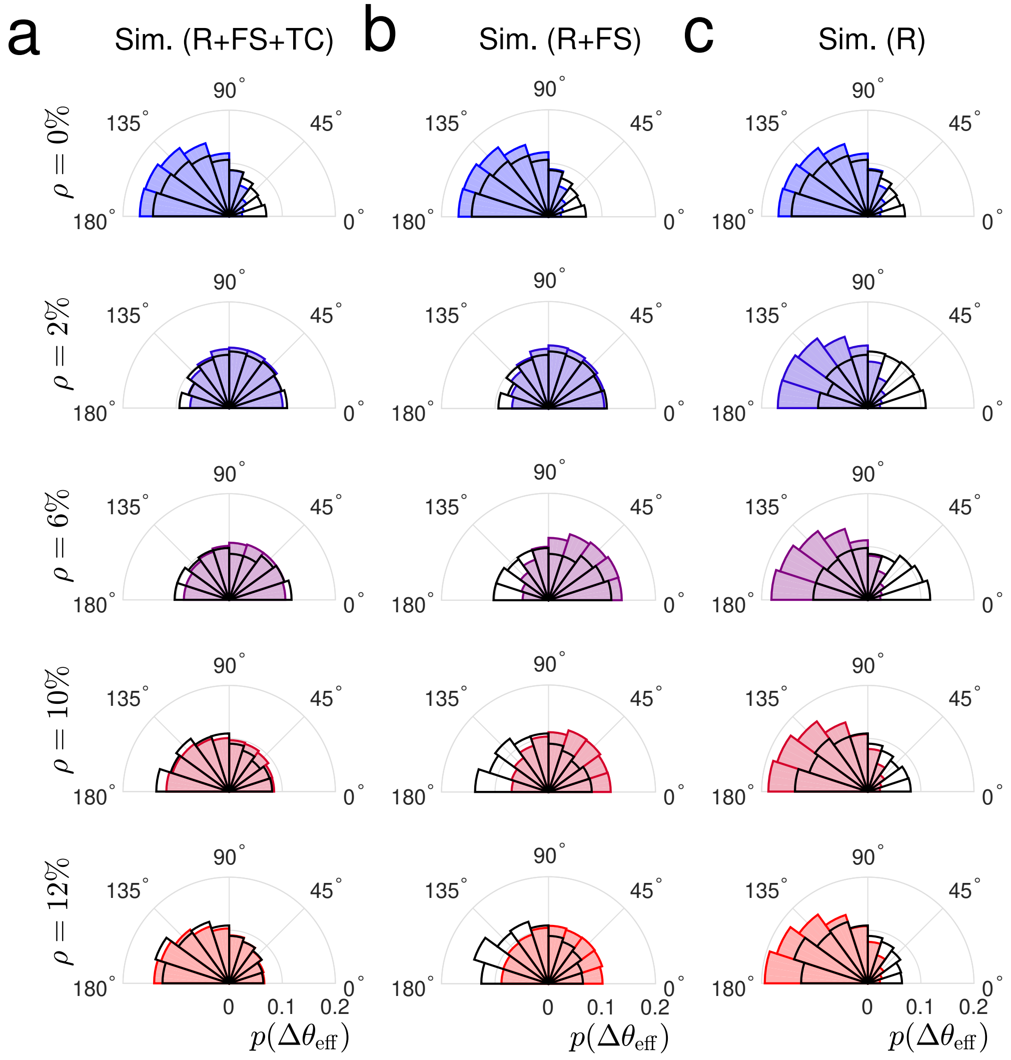

Supplementary Figure 6. **Simulated change in effective propagation direction for chiral active particles in the presence of randomly distributed micro-obstacles.** Probability distributions of the change in effective propagation direction $\Delta\theta_{\text{eff}}$ for simulated chiral active particles self-propelling through a circular area of radius $R = 25\,\mu\text{m}$ containing obstacles distributed at random without overlap (Supplementary Fig. **5**a). The particles self-propel in the presence of (**a**) all three cell-obstacle interaction terms (R: repulsive interaction; FS: forward scattering; TC: tumble-collisions), (**b**) without tumble-collisions (TC) and (**c**) with repulsion (R) alone (Methods). The probability distributions are shown for different obstacle densities ρ: $\rho = 0\%$, $\rho = 2\%$, $\rho = 6\%$, $\rho = 10\%$ and $\rho = 12\%$. Each distribution is obtained from 3000 different trajectories. $\Delta\theta_{\text{eff}} = 90°$ separates between forward ($\Delta\theta_{\text{eff}} < 90°$) and backward ($\Delta\theta_{\text{eff}} > 90°$) propagation. For reference, the corresponding experimental distributions for the different values of ρ (Fig. 2) are shown as solid black lines.

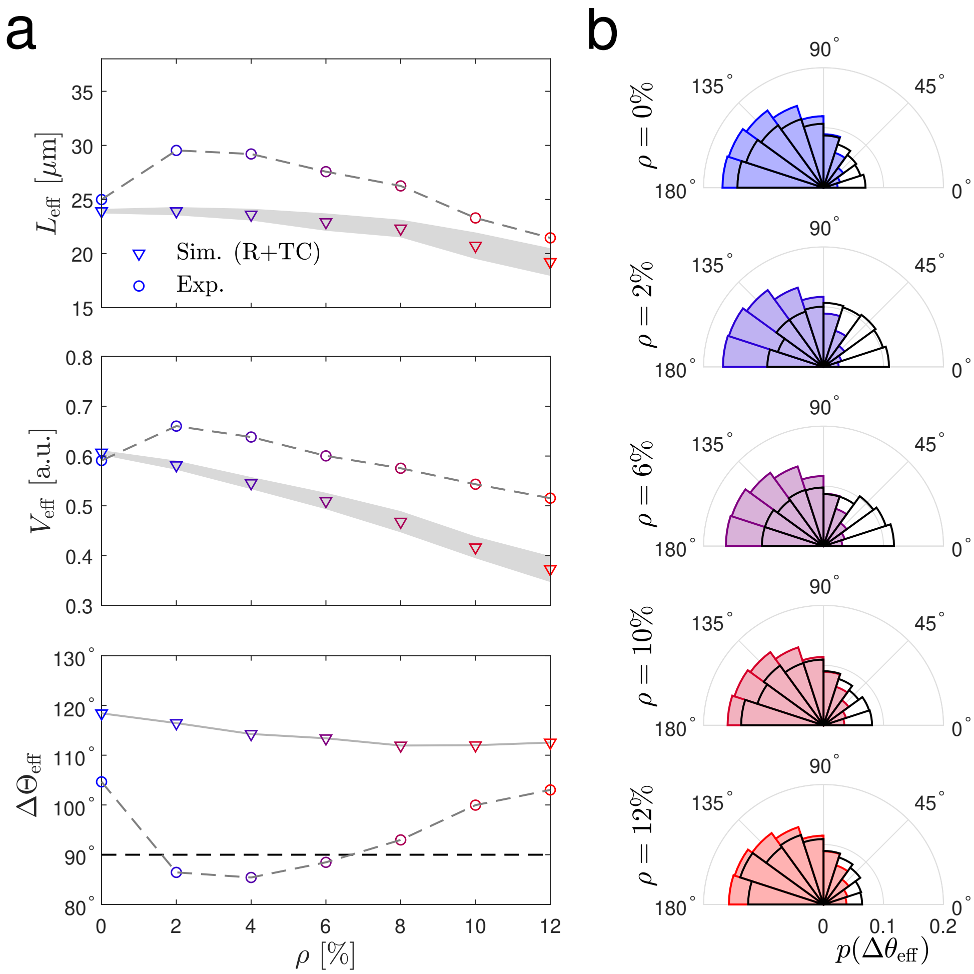

Supplementary Figure 7. **Simulations including repulsion and tumble-collisions alone.** (**a**) Simulated average effective propagation distance L_{eff}, normalised average effective propagation speed V_{eff} and average change in effective propagation direction $\Delta\Theta_{\mathrm{eff}}$ as a function of the obstacle density ρ for chiral active particles self-propelling through a circular area of radius $R = 25\,\mu$m containing obstacles distributed at random without overlap (Supplementary Fig. **5**a). The particles self-propel in the presence of repulsive interactions (R) and tumble-collisions (TC) alone (Methods). Each value is obtained from averaging over 3000 different trajectories. The shaded area around the mean values of L_{eff} and V_{eff} represents one standard deviation. The solid line connecting the values of $\Delta\Theta_{\mathrm{eff}}$ is a guide for the eyes. The corresponding experimental values (Figs. 1 and 2) are shown for reference (circles). (**b**) Corresponding probability distributions of the change in effective propagation direction $\Delta\theta_{\mathrm{eff}}$. The probability distributions are shown for different obstacle densities ρ: $\rho = 0\%$, $\rho = 2\%$, $\rho = 6\%$, $\rho = 10\%$ and $\rho = 12\%$. $\Delta\theta_{\mathrm{eff}} = 90^{\circ}$ separates between forward ($\Delta\theta_{\mathrm{eff}} < 90^{\circ}$) and backward ($\Delta\theta_{\mathrm{eff}} > 90^{\circ}$) propagation. For reference, the corresponding experimental distributions for the different values of ρ (Fig. 2) are shown as solid black lines.

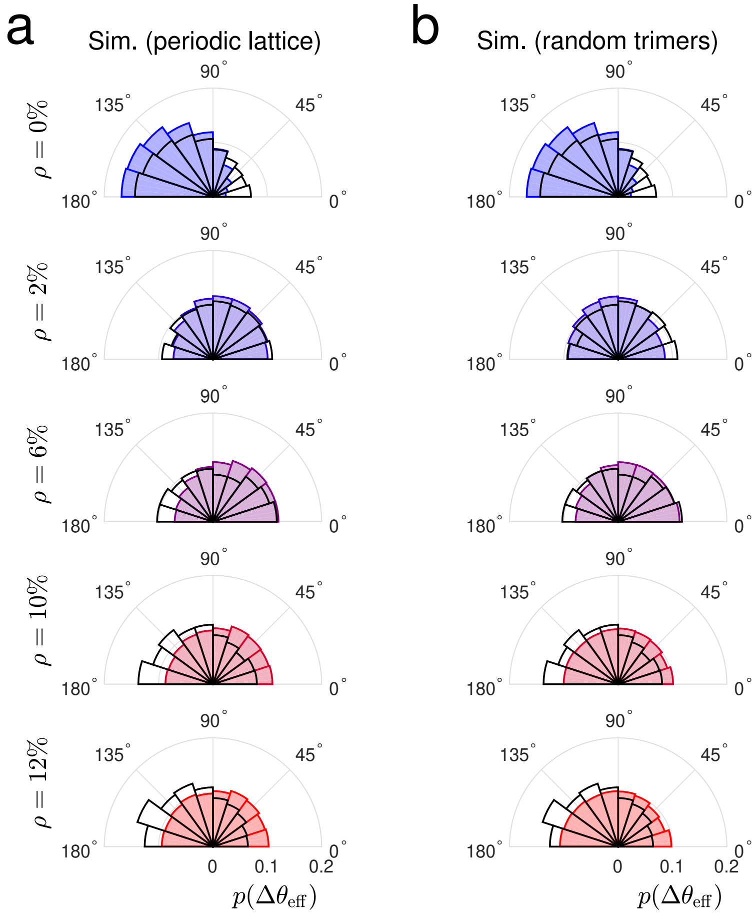

Supplementary Figure 8. Simulated change in effective propagation direction for chiral active particles in the presence of micro-obstacles with different distributions. Probability distributions of the change in effective propagation direction $\Delta\theta_{\rm eff}$ for simulated chiral active particles self-propelling through a circular area of radius $R = 25\,\mu$m containing obstacles distributed according to (**a**) a triangular periodic lattice (Supplementary Fig. **5**b and Methods) and (**b**) a random distribution of non-overlapping trimers (Supplementary Fig. **5**c and Methods). The interactions with the obstacles include all three cell-obstacle interaction terms: repulsive interactions, forward-scattering events, and tumble-collisions (Methods). The probability distributions are shown for different obstacle densities ρ: $\rho = 0\%$, $\rho = 2\%$, $\rho = 6\%$, $\rho = 10\%$ and $\rho = 12\%$. Each distribution is obtained from 3000 different trajectories. $\Delta\theta_{\rm eff} = 90°$ separates between forward ($\Delta\theta_{\rm eff} < 90°$) and backward ($\Delta\theta_{\rm eff} > 90°$) propagation. For reference, the corresponding experimental distributions for the different values of ρ (Fig. 2) are shown as solid black lines.

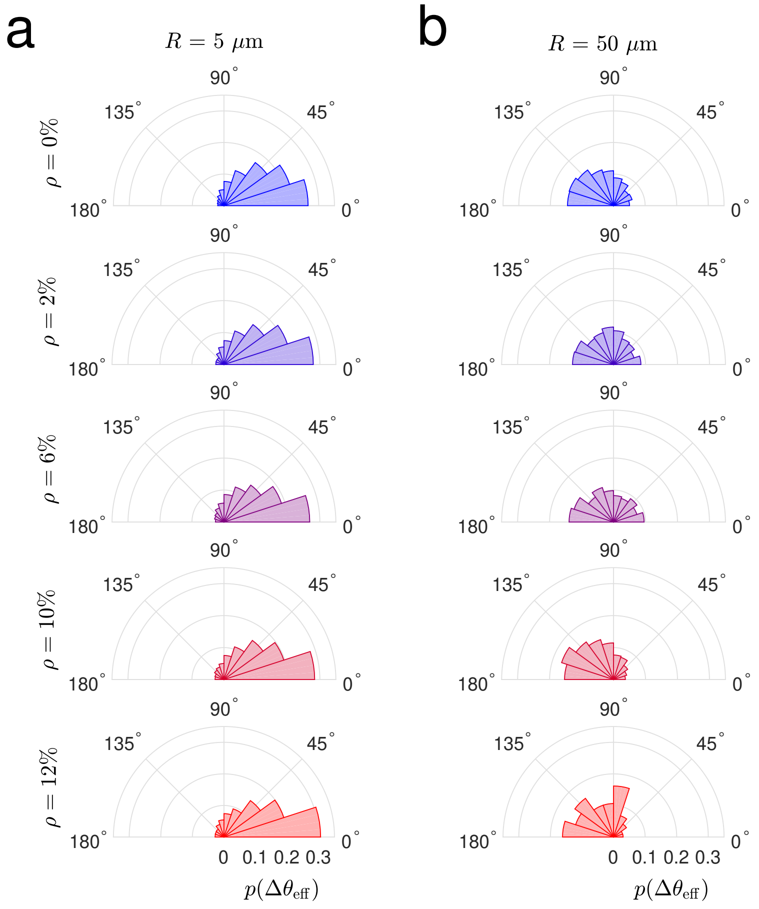

Supplementary Figure 9. **Change in effective propagation direction for *E. coli* cells swimming through circular areas of different radii.** (**a-b**) Probability distributions of the change in effective propagation direction $\Delta\theta_{\mathrm{eff}}$ for *E. coli* cells swimming through a circular area of (**a**) $R = 5\,\mu$m and (**b**) $R = 50\,\mu$m for different obstacle densities ρ: $\rho = 0\%$, $\rho = 2\%$, $\rho = 6\%$, $\rho = 10\%$ and $\rho = 12\%$. Each distribution is obtained from at least 200 different trajectories. $\Delta\theta_{\mathrm{eff}} = 90°$ separates between forward ($\Delta\theta_{\mathrm{eff}} < 90°$) and backward ($\Delta\theta_{\mathrm{eff}} > 90°$) propagation.

SUPPLEMENTARY REFERENCES

[1] Masson, J.-B., Voisinne, G., Wong-Ng, J., Celani, A. & Vergassola, M. Noninvasive inference of the molecular chemotactic response using bacterial trajectories. *Proc. Natl. Acad. Sci. U.S.A.* **109**, 1802–1807 (2012).
